# Diagnostic performance of chest CT findings of COVID-19 with RT-PCR negative

**DOI:** 10.4314/ahs.v22i4.56

**Published:** 2022-12

**Authors:** Soukaina Wakrim

**Affiliations:** Ibn Zohr University, Department of Radiology, University Hospital of Agadir, Faculty of Medicine and Pharmacy

**Keywords:** COVID-19, Tomography, Spiral Computed, COVID19 serological testing, COVID-19 diagnostic testing, Thorax

## Abstract

COVID-19 presented with lung abnormalities on computed tomography (CT) scans in patient with false negative RT-PCR, which are helpful in diagnosis of this emerging global health emergency. It's a case report the young woman of 35-year-old patient with 2019-nCoV pneumonia confirmed with IgM-IgG serology underwent thin-section Chest CT.

Our patient has the Chest CT with some lung abnormalities, the Ground-glass opacities, crazy paving pattern and smooth interlobular septal thickening. The clinical findings and with conspicuous ground grass opacity lesions in the peripheral and posterior lungs on CT are highly suspected of 2019-nCoV pneumonia.

## Introduction

The 2019 novel coronavirus (2019-nCoV or officially named by the World Health Organization as COVID-19) is a viral respiratory illness, was firstly reported in Wuhan, Hubei Province, China, and rapidly spreads as a pandemic^1^. As for the rest of the world, my country is one of the countries actively fighting the COVID-19 pandemic. Here, we report chest CT findings in one patient confirmed with 2019-nCoV pneumonia in IgM-IgG serology at our hospital, with false negative RT-PCR.

## Case report

A 35 years old woman have just given birth by C-section, she was admitted to the emergency department, with fever, coughs and throat discomfort for 2 days. She had a history of good physical health and no underlying diseases. Physical examination showed fever, with a body temperature of 38.7°C, and the laboratory examination results showed abnormal leukocyte count, increased neutrophils, decreased lymphocytes, and elevated C-reactive protein.

**Figure 1:**
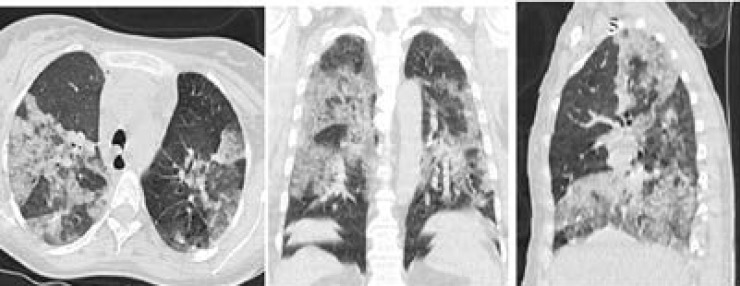
Initial thin-section non contrast Chest CT scan were performed on day of admission (from 2/4/2020): Axial, coronal and sagittal planes show multiple ground-glass opacities and a crazy paving pattern in bilateral lung.

The first thin-section non contrast Chest CT scan from 2/4/2020 shows multiple ground-glass opacities and a crazy paving pattern in bilateral lung. The bilateratism of the peripheral lung opacities, without subpleural sparing, are common CT finding of the 2019 novel coronavirus pneumonia (figure 1). The patient tested negative for 2019-nCoV based on the real-time reverse-transcriptase-polymerase chain reaction (rRT-PCR) amplification of the viral DNA from a nasal swab.

**Figure 2:**
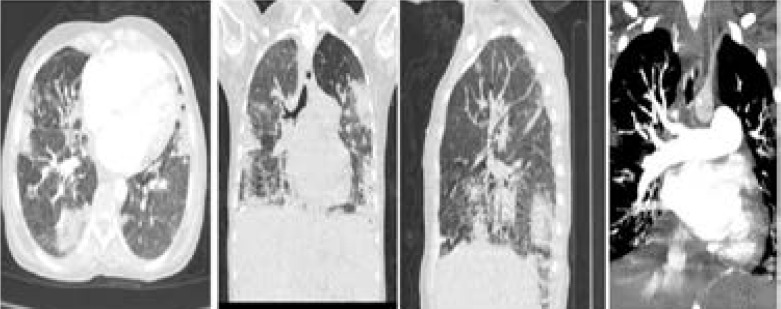
Follow-up Axial, coronal and sagittal planes of thin-section non-contrast CT image in pulmonary parenchymal taken 6 days after (From 8/4/2020), show increased density of GGOs which then progressed into consolidations with perilobular thickening. Chest CT with contrast at coronal plane in mediastinal-window, show neither pulmonary embolism.

In the presence of the manifested clinical progression and the worsening symptoms, the second Thin-section none and with contrast Chest CT scan from 8/4/2020 (6 days after follow-up) shows progressive ground-glass opacities and multiple regions of patchy consolidation in two lungs with neither cardiopulmonary abnormalities such as pulmonary embolism (figure 2). Following this CT scan, another reverse-transcriptase-polymerase chain reaction (rRT-PCR) from a nasal swab was performed and it was negative. But She was diagnosed with 2019-nCoV based on the IgM-IgG serology.

**Figure 3:**
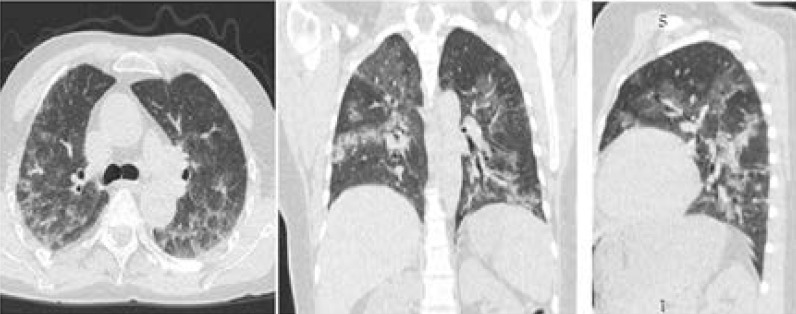
13 days after follow-up (21/4/2020), Chest CT non-contrast in axial, coronal and sagittal pulmonary parenchymal show reduced size and density of the lesions.

The patient received a hydroxychloroquine with the azithromycin leading to increased benefit and good clinical course. 13 days after follow-up, CT showed regression of patchy consolidation and ground-glass opacities in both lungs with a right pleural effusion (figure 3).

## Discussion

In December 2019, the Novel coronavirus (2019 -nCOV) is identified in Wuhan, China, which causes a new type of pneumonia. Since then, the COVID- 19 is a respiratory pandemic disease 2,3.

The WHO has declared SARS-CoV-2 a pandemic, because the rapid rise in number of cases; uncontrolled and vast worldwide spread 4.

The clinical presentation of COVID-19 can from being asymptomatic to being in severe acute respiratory distress. The real-time polymerase chain reaction (RT-PCR) is method of diagnostic the COVID-19 to detect viral nucleotides from specimens obtained by oropharyngeal swab, nasopharyngeal swab, bronchoalveolar lavage, or tracheal aspirate^4^. Nevertheless, recent publication have result that RT-PCR has sensitivity as low as 60–71% for detecting COVID-19^5^, ^6^, in relation either laboratory error or low viral load present in test specimens^7^, ^8^.

Therefore, these false negatives caused repeat testing and critical resource constraints^8^.

Some studies have concluded that serologic assays are crucial method to identified the viral reservoir hosts and for epidemiological studies, as well as to reveal the forms of asymptomatic infections and to evaluate the morbidity and mortality.

Generally, imaging is indicated in a patient with COVID-19 and worsening respiratory status, based essentially for Computed tomography (CT) which is key tools for pulmonary disease diagnosis and management^3^.

On the one hand, Chest CT non-contrast has demonstrated about 56–98% sensitivity in differentiating COVID-19 from viral pneumonia, on the other hand it can be useful in early stages of disease course to detect this pathology with false negatives obtained from RT-PCR^7^, ^9^.

The initial Thin-section non contrast Chest CT of COVID-19 reveals areas of “crazy-paving” patterns and ground-glass opacity (GGO) peripheral, developed in subpleural areas in bilateral lung. In Follow-up, the GGO it was increased of density and size in multiple lobes progressing to consolidation. CT signs gradually improve approximately 14 days post-symptom onset 10–11.

## Conclusion

A diagnosis is made considering clinical findings, epidemiology, and laboratory testing to confirm SARS-CoV-2 infection. Chest CT can be a pivotal diagnostic role in the assessment of patients with COVID-19.
